# Perceptions of Telehealth vs In-Person Visits Among Older Adults With Advanced Kidney Disease, Care Partners, and Clinicians

**DOI:** 10.1001/jamanetworkopen.2021.37193

**Published:** 2021-12-06

**Authors:** Keren Ladin, Thalia Porteny, Julia M. Perugini, Kristina M. Gonzales, Kate E. Aufort, Sarah K. Levine, John B. Wong, Tamara Isakova, Dena Rifkin, Elisa J. Gordon, Ana Rossi, Susan Koch-Weser, Daniel E. Weiner

**Affiliations:** 1 Research on Ethics, Aging, and Community Health (REACH Lab), Medford, Massachusetts; 2 Departments of Occupational Therapy and Community Health, Tufts University, Medford, Massachusetts; 3 William B. Schwartz MD Division of Nephrology, Tufts Medical Center, Boston, Massachusetts; 4 Department of Medicine, Tufts Medical Center, Boston, Massachusetts; 5 Center for Translational Metabolism and Health, Institute for Public Health and Medicine, Northwestern University Feinberg School of Medicine, Chicago, Illinois; 6 Division of Nephrology, Veterans’ Affairs Healthcare System, San Diego, California; 7 University of California, San Diego; 8 Department of Surgery-Division of Transplantation, and Center for Health Services and Outcomes Research, and Center for Bioethics and Humanities, Northwestern University Feinberg School of Medicine, Chicago, Illinois; 9 Piedmont Transplant Institute, Atlanta, Georgia; 10 Department of Public Health & Community Medicine, Tufts University School of Medicine, Massachusetts

## Abstract

**Importance:**

Telehealth has been posited as a cost-effective means for improving access to care for persons with chronic conditions, including kidney disease. Perceptions of telehealth among older patients with chronic illness, their care partners, and clinicians are largely unknown but are critical to successful telehealth use and expansion efforts.

**Objective:**

To identify patient, care partner, and nephrologists’ perceptions of the patient-centeredness, benefits, drawbacks of telehealth compared to in-person visits.

**Design, Setting, and Participants:**

This qualitative study used semistructured interviews conducted from August to December 2020 with purposively sampled patients (aged 70 years or older, chronic kidney disease stages 4 to 5), care partners, and clinicians in Boston, Massachusetts; Chicago, Illinois; Portland, Maine; and San Diego, California.

**Main Outcomes and Measures:**

Participants described telehealth experiences, including factors contributing to and impeding engagement, satisfaction, and quality of care. Thematic analysis was used to analyze data.

**Results:**

Of 60 interviews, 19 (32%) were with clinicians, 30 (50%) with patients, and 11 (18%) with care partners; 16 clinicians (84%) were nephrologists; 17 patient participants (43%) were non-Hispanic Black, and 38 (67%) were women. Four overarching themes characterized telehealth’s benefits and drawbacks for patient-centered care among older, chronically ill adults: inconsistent quality of care, patient experience and engagement, loss of connection and mistrust (eg, challenges discussing bad news), and disparities with accessing telehealth. Although telehealth improved convenience and care partner engagement, participants expressed concerns about clinical effectiveness and limitations of virtual physical examinations and potentially widening disparities in access. Many participants shared concerns about harms to the patient-clinician relationship, limited ability to comfort patients in virtual settings, and reduced patient trust.

**Conclusions and Relevance:**

Older patients, care partners, and kidney clinicians (ie, nephrologists and physician assistants) shared divergent views of patient-centered telehealth care, especially its clinical effectiveness, patient experience, access to care, and clinician-patient relationship. Understanding older patients’ and kidney clinicians’ perceptions of telehealth elucidate barriers that should be addressed to promote high-quality care and telehealth use.

## Key Points

**Question:**

How are telehealth encounters perceived by older adults with chronic kidney disease, their care partners, and their clinicians?

**Findings:**

In this qualitative study, involving 60 interviews conducted during 2020, lower satisfaction was associated with telehealth among older patients with lower socioeconomic status and among patients identifying as Black, Hispanic, and Native American. Drawbacks to telehealth included care quality concerns because of limited physical examination and laboratory tests and loss of social connection, while benefits included convenience, greater care partner engagement, and clinicians’ understanding of patients’ home environment.

**Meaning:**

These findings suggest that while telehealth reduces barriers to care for some older adults, greater resources are needed to support many older adults with chronic illness, including limited English proficiency, hearing loss, and those with limited access to internet and technology.

## Teaser

This qualitative study identifies patient, care partner, and clinicians’ perceptions of the patient-centeredness, benefits, and drawbacks of telehealth compared with in-person visits.

## Introduction

Telehealth use increased dramatically during the COVID-19 pandemic among older adults with chronic conditions, including chronic kidney disease (CKD).^1^ Approximately 38% of US adults over 65 years have CKD, corresponding to 20% of traditional Medicare payments (ie, $114 billion annually).^2^ Telehealth is likely to remain significant to CKD care, bolstered by the Advancing American Kidney Health Initiative and the 2018 Bipartisan Budget Act.^3,4^ Even before the COVID-19 pandemic, telehealth was seen as a cost-effective strategy to improve access to care for patients with chronic illnesses.^5,6,7^ Yet little is known about whether and how telehealth promotes patient-centered care among older adults with complex conditions, such as CKD.

Telehealth may improve access to patient-centered care, particularly for populations facing challenges with transportation or caregiving, and those seeking to distance during the pandemic.^8,9,10^ Some patients with CKD appear willing to use telehealth,^11,12^ and outcomes appear similar between those receiving telehealth vs in-person care in small, observational reports.^13,14^ Surveys suggest that many patients with chronic conditions are satisfied with telehealth care,^14,15,16^ and telehealth has been successfully used for CKD disease management.^9,17^

Telehealth may have drawbacks, particularly among older adults with chronic conditions. Older adults commonly have less access to and less comfort with technology than younger adults.^18^ Moreover, telehealth may compromise care quality because of inadequate physical examinations and laboratory tests, key care components.^19,20,21^ Some studies demonstrate lower telehealth willingness and satisfaction among persons who are older, identify as Black, or have less education.^22^ The impact of telehealth on engagement and experience of older, chronically ill adults remain insufficiently understood.

Most studies involving patients have relied on surveys or claims data, which, although instructive, are insufficient for understanding telehealth experiences of older persons with chronic conditions.^11,19,23,24,25^ This qualitative study conducted at 4 sites uniquely addresses these gaps by identifying factors affecting telehealth experiences of older adults with CKD, their care partners, and kidney clinicians to improve patient-centered telehealth.

## Methods

This qualitative study was approved by the institutional review board at Tufts University School of Medicine. Phone interviews were conducted and recorded, and notes were taken by trained researchers (T.P., K.G.) from August to December 2020 following verbal informed consent, where study goals and rationale was explained. This study followed the Consolidated Criteria for Reporting Qualitative Research (COREQ) reporting guideline.^26^

This qualitative study is part of the Decision Aid for Renal Therapy (DART) Trial (ClinicalTrials.gov NCT03522740), which examines the effectiveness of a decision aid to support older patients facing dialysis decisions (eAppendix 1 in the Supplement). The DART Trial recruited 400 English-fluent patients age 70 years or older with nondialysis CKD and an estimated glomerular filtration rate less than 30mL/min/1.73 m^2^ receiving care at nephrology clinics in Greater Boston, Massachusetts; Portland, Maine; San Diego, California; and Chicago, Illinois. This subsample was selected from participants who were followed up with for 12 months, following purposive sampling criteria, including age, gender, race, ethnicity, treatment preference, and site.

Patients self-identified race and ethnicity using US census categories. Race was included because prior literature suggests that there may be disparities in access to and experiences with telehealth by race and ethnicity, and we felt it was important to more deeply understand this. Additionally, our stakeholder advisory board, which is comprised of individuals of diverse expertise (eg, patients, caregivers, policy makers, patient organizations, clinicians, etc.) and racial and ethnic diversity, strongly recommended that we examine this.

### Data Collection

A social scientist with expertise in qualitative methods and kidney disease (K.L.)^27,28^ designed 3 semistructured interview guides based on literature review and clinical experience (ie, patients, care partners, clinicians). The guides were refined by the research team and the DART Stakeholder Advisory Board (eAppendix 1 in the Supplement). We recruited a subset of DART participants following purposive sampling criteria: gender, age, race and ethnicity, region, and education (patients), and years in practice (clinicians). Care partners, nominated by patients as the people with whom they discuss health care decisions, were approached and consented independently. Clinicians were recruited via email. Open-ended questions explored patients’, care partners’, and clinicians’ perceptions of telehealth and patient-centered care implications (eAppendix 2 in the Supplement).

### Data Analysis

Interviews were transcribed verbatim and continued until thematic, and sampling saturation was achieved and confirmed through deliberation.^29^ The team developed a preliminary codebook for patient and care partners and another for clinicians, following interview guides. At least 4 researchers (T.P., K.G., K.A., J.P.) independently coded 10 interviews line by line, allowing for new codes to emerge inductively using NVivo version 11 (QSR International). Iterative deliberation produced a consensus about coding discrepancies and emergent codes.^30^ The team refined the codebook and independently recoded the original 10 interviews and 6 additional transcripts. Codes were clarified and organized into categories through a consensus process to encompass the range and variability of subthemes and to characterize confirmatory and contradictory narratives.^31^ Emerging themes were discussed and refined by the research team. Following thematic analysis, researchers further analyzed commonalities and differences in opinions and experiences of telehealth by patient self-reported race and ethnicity.

## Results

We conducted 60 interviews (19 [32%] clinicians; 30 [50%] patients, and 11 [18%] care partners); of the patients, 13 (43%) were non-Hispanic Black participants, 20 (67%) were women, and 22 (73%) were 75 years or older. Sixteen clinicians (84%) were nephrologists (Table 1). The mean (SD) interview time was 30 (11) minutes.

**Table 1.** Sample Characteristics

Characteristic	No. (%)		
	Clinician (N = 19)	Patient (N = 30)	Care partner (N = 11)^a^
Gender			
Woman	10 (53)	20 (67)	8 (73)
Man	9 (47)	10 (33)	3 (27)
Age, y			
40-49	NA	NA	1 (9)
50-59	NA	NA	2 (18)
60-69	NA	NA	2 (18)
70-74	NA	8 (27)	2 (18)
75-79	NA	13 (43)	1 (9)
≥80	NA	9 (30)	3 (27)
Clinician type			
Nephrologist	16 (84)	NA	NA
Physician Assistant	3 (16)	NA	NA
Region			
Boston	7 (37)	6 (20)	2 (18)
Chicago	6 (32)	11 (37)	5 (45)
San Diego	5 (26)	12 (40)	4 (36)
Maine	1 (5)	1 (3)	0 (0)
Race^b^			
White	NA	13 (43)	7 (64)
African American/Black	NA	13 (43)	4 (36)
Other	NA	4 (13)	NA
Ethnicity^b^			
Hispanic/Latino	NA	2 (7)	1 (9)
Education level			
High school	NA	5 (17)	1 (9)
Some college or technical school	NA	12 (40)	3 (27)
College graduate	NA	4 (13)	3 (27)
Post-graduate	NA	9 (30)	4 (36)
Care partner status			
Has a care partner	NA	13 (43)	NA

Abbreviations: DART, Decision Aid for Renal Therapy (DART) Trial; NA, not applicable.

^a^ Patient has a care partner enrolled in the DART Trial.

^b^ Participants self-identified their race and ethnicity from the following categories: White, Black or African American, Asian, Native Hawaiian or other Pacific Islander, Native American or Alaskan Native, Other, Don’t Know, or Refused. Of the 4 participants categorized as other in this Table, 2 selected Other, 1 selected Asian, and 1 selected Native American. Participants were also able to choose multiple categories.

### Variable Quality of Care

Most clinicians viewed telehealth as compromising quality care due to an inability to conduct physical examinations and laboratory tests, including inaccurate edema and blood pressure measurement (Table 2). For example, clinician 18 shared: “It’s not good medicine to…not see people in person. The physical exam is really a part of what we do and not being able to examine the patient is a problem.” Clinician 2 said: “I’m always worried I’m missing something.” Clinician 1 said: “Our [telehealth] assessment is not as good. There’s a lot of holes. You’re going to get in trouble if you do [telehealth] too many times sequentially.” Clinician 15 stated: “I just really hate [telehealth]…So it saves maybe a bit of [COVID-19] exposure, but unfortunately [we] need labs. I don’t know how much we’re really saving them in terms of keeping them safe” Clinicians also noted difficulty transitioning rapidly to virtual visits because of inadequate support.

**Table 2.** Variability of Quality of Care

Participant	Example responses
Clinician	“I find [telehealth] very challenging to actually...If I have someone who's ill, it's very limited. I can't examine them. I can't even check their blood pressure. I can't check if they have swelling. I can't listen to them. I'm usually telling them, ‘You know what, I need to see you. Come in next week, and we'll actually get you in the office.’ It's challenging for someone who is ill.” (Clinician 2)
Care partner	“[T]hen she had to do blood work. She can't do that over the phone. She had to do a CAT scan and stuff like that.” (Care partner 9)
Patient	“The problem [with telehealth] is just that I don't get enough teaching in my diseases.” (Patient 19)

Still, a few clinicians valued the holistic understanding of patients’ home environment and self-management. For example, clinician 6 said: “When you’re going through the medications, [patients]…can actually hold the bottle up and show you. There are some things that you can do better [with telehealth]. It does give you a little bit of a view of the setting in which the patient lives” Some clinicians said telehealth empowered patients to participate in their care. For example, clinician 14 said: “We’re learning [patients are] able to check their blood pressures, knowing that they can show me…[what] I need in order to make a good assessment.”

Patients worried about quality care and home diagnostics. Patient 33 shared: “I don't think it’s a good idea to try to diagnose people over the telephone…Your machine may not be as good as the ones at the doctor’s office, and you may be getting a wrong result.” Patient 7 said: “I'm not good at doing the blood pressure at home”

### Patient Engagement With Clinicians

Patients, care partners, and clinicians described telehealth as more convenient, less costly, and more efficient for patients than clinic visits (Table 3). Patient 15 explained: “I actually prefer [telehealth] rather than have to hike…to the medical center.” Most clinicians felt that telehealth was very convenient and that patients did not have to spend time in their car or waiting. For example, clinician 11 said: “[Telehealth] is very convenient….[Patients] don’t need to spend their time in the car or waiting.” Clinician 13 said: “I can also see patients off my regular clinic hours. I think my availability to patients has actually improved compared to before.”

**Table 3.** Patient Experience and Engagement With the Health System

Participant	Example responses
**Positive Experience**	
Clinician	“Patients feel that they’re getting the attention that they need without [the] risk.” (Clinician 14)
Care partner	“We do it by telephone…and I have participated.” (Care partner 13)
Patient	“There wasn't anything that made [telehealth] difficult. I was comfortable with it, I understood what the purpose was.” (Patient 14) “Instead of coming home from the doctor's office or the hospital and going, ‘Now what did they tell me to do? Who did I see?’ It's all written out for you after visit in telehealth. I have it there forever. It's in my file now. It's written out.” (Patient 29)
**Challenging Experience**	
Clinician	“[The] problem is some patients really don’t have a space for themselves. They have to go outside…they have people around them, not a very private environment.” (Clinician 8) “It's been pretty challenging....We've had a lot of technical difficulties along the way. It's rare that you do an entire half day of clinic without at least one person that you're not able to connect with. Either they can't hear you or they can't see you, or they are not able to log on at the time of the appointment and you end up switching to a phone visit. Or even on the phone sometimes you cannot hear each other, It's been challenging because then you've spent, out of the 20 minutes you have together, you spend 10 minutes trying to connect and it's been quite frustrating.” (Clinician 18)
Patient	“I just think the communication is better [in-person]. On the telephone, I probably don't [ask] any questions anyway. I just listen.” (Patient 21)
Care partner	NA

Patients appreciated the relaxed home environment and reducing COVID-19 risks. Patient 8 said: “You’re more comfortable when you’re not sitting on a stool in some doctor’s office waiting room. You don’t have to dress up.” Telehealth also facilitated care partner engagement especially while visitation restrictions were in place. Care partner 17 explained: “If [I] wasn’t at the appointment, then I would worry, ‘Did [they] tell her everything?’ [Telehealth] has been very helpful.” Patient 23 explained: “My sister’s got a computer.…She contacts the doctors, it's working out wonderful.” Clinicians emphasized the importance of care partner participation; for example, clinician 7 stated that “What was relevant to telehealth is that the patient might have a relative at home with them [making] the logistics [are] a lot easier.”

However, technical challenges and a lack of a quiet, private place limited patient engagement, with few engaging meaningfully in discussion. Clinician 16 noted that: “The technology’s not perfect. Sometimes there are sound issues…internet connection issues. There’s not really a lot of real-time help for the patient.” Another clinician noted that there were “lots of technical challenges...which are dumped on the provider and the patient and which we have to solve without a lot of technical support.” Patient 17 expressed frustration and said: “We attempted the video, but I was not sophisticated enough…to get it to work.”

Clinicians further attributed poor patient engagement to telehealth’s lack of formality. Clinician 19 said: “There is kind of less formality…but these are supposed to be appointments where you have someone’s undivided attention.” Clinician 6 recalled a televisit in which “[the patient] was walking through the city and we were getting seasick watching her head bounce up and down and the sky shift and the buildings shift behind her.” Clinicians also cited more missed appointments. For example, clinician 7 remarked that it was “easier for patients to just not show up for a phone call, or Zoom, than to the office.” Regarding scheduling problems, clinician 5 said: “There [are scheduling] screw-ups…[Patient’s] waiting for a call and if they don’t get it, they’re sort of upset by that.”

### Loss of Interpersonal Connection and Mistrust

Patients and clinicians bemoaned loss of social connection (Table 4). Patient 20 said: “I don’t get a sense of the doctor as a person, [only as a] strict clinician with no real connection with me. But in the office, it’s different.” Another patient said: “[In person], I can feel the vibe whether you’re giving a damn or just going through the motions…I really wish I had had a chance to meet [the doctor].” Even patients with established relationships struggled; for example, patient 12 said: “Not only do I like seeing my doctors medically, I like [talking] to them and hear what’s going on with their lives.” Yet other patients disagreed. Patient 4 stated:“On the phone, [clinicians are] more tender than they are in person.” Patient 36 thought that “maybe the doctor is more committed to providing his time because of [telehealth]…we’re able to have a greater dialogue…than at the office.”

**Table 4.** Loss of Connection and Mistrust (Challenges Discussing Bad News)

Participant	Example responses
**Loss of Connection and Mistrust**	
Clinicians	“It's been a little bit challenging not seeing people in person. Because I think that it makes it harder to... really get a sense of what the patient is thinking or answer questions they have...I think [in-person visit] makes it easier to read the patient's reaction.” (Clinician 18) “I think you're losing a lot of the multi-dimensionality….When you're just doing it by a screen, you're not seeing body language necessarily, you're not aware of eye contact, you're not aware of other people in the room and their reactions. I find you lose a lot of sort of those additional clues as to how things are going and the reaction of the person.” (Clinician 1) “It's convenient, but I think you miss out a lot. Sometimes…the connection is lost, that sort of human connection. I find it difficult to get a sense of how well they're doing. It's easy to smile on camera and say, ‘Everything's fine.’ You just don't get a good sense of how people are doing so I'm making more and more people come in.” (Clinician 8) “Talking about major issues in their health and trying to garner [patients’] trust and appeal to them, sometimes can feel a little bit... distant. I think it's more powerful in person, usually.” (Clinician 19)
Patients	“There's always an occasional technical glitch. You can't connect for some reason. All this technology, it's basically dehumanizing us. I think it does take away…the personal contact. I do miss that. Especially meeting with the staff.” (Patient 35) “Not only do I like seeing my doctors medically, I like [talking] to them and hear what’s going on with their lives.” (Patient 12)
**Challenges discussing bad news**	
Clinicians	“Much more [difficult with] telehealth because you're not even there physically in the same space, which you can't even hand them a tissue box or something.” (Clinician 13) “I just had a conversation with this 22-year-old about her life expectancy over the phone. That was not fun. I try to save it for in-person.” (Clinician 8)

Clinicians cited loss of connection and difficulty understanding patients’ feelings. For example, clinician 12 stated: “I do think that there’s a loss of connection that occurs across a screen.” Others, such as clinician 13, described mistrust among older patients: “The elderly, who are very skeptical…have that notion that a visit with the doctor has to be in person.” Other clinicians noted that difficulty with listening contributed to lower quality conversations. Clinician 1 remarked: “I’m less good at listening with telemedicine than I would be in the office. Certainly, less good at assessing feedback and recognizing if I said something and the patient had a reaction to.”

### Difficulty of Breaking Bad News

Clinicians struggled understanding patient emotions and being sufficiently empathic virtually. For example, clinician 1 stated: “You need to be in the room with someone to see how they’re reacting. I never felt at all comfortable with these types of heavy decisions being made remotely” Clinician 13 clarified: “You’re not even there physically in the same space, which you can’t even hand them a tissue box or something.”

Some clinicians employed strategies, such as relying on telehealth only with established patients or writing detailed summaries. Clinician 5 said: “If I know [patients] well… it’s much easier to have those types of conversations [via telehealth].” Clinician 3 stated: “If [patient] is in our [electronic medical records (EMR)], I will say: ‘Look, I’m going to summarize what I said. I’m going to send you a message. If you have any questions, reply to me.’ If it’s a heavy conversation and they’re not on the EMR, that’s harder.” Others encouraged follow-up by advanced care practitioners or tried simulating the clinic experience. For example, clinician 16 said: “I just try to pause and listen and not rush the patient, even if it means I’m being late for the next patient. I try to simulate …clinic, the only thing that’s missing [is] Puffs or Kleenex, but I can’t offer that.”

### Disparities Accessing Telehealth

Clinicians reported that older patients and patients from structurally underserved populations (eg, patients with low socioeconomic status, limited health literacy, hearing impairment, or non-English speaking) had poorer telehealth experiences (Table 5). For example, clinician 5 stated: “Some older patients may not be able to get video calls working or may not have the technical capability. That makes the quality of visits much less.” Others noted socioeconomic disparities limiting patient engagement, including clinician 19 who said: “those who don’t have the means to have video chat capability on their phone, or don’t know how to activate that.” Clinician 5 made a suggestion regarding access and said: “More people need to have [access]…that may mean…getting [them] a fairly inexpensive tablet…for telehealth.”

**Table 5.** Theme 4: Disparities With Accessing Telehealth

Disparities	Example responses
**Clinicians**	
Language-based disparities	“I do think it makes...I think it is more difficult with the interpreter for those that don't speak English, no matter what the language is. We still have interpreter services, but I feel like a lot more is a loss for those patients, versus in-person, where they see me speaking more clearly, my body language.” (Clinician 16)
Hearing loss disparities	“I had a woman who was really hard of hearing and I had the hardest time because her husband usually listens for her [and was unavailable]. [For] some patients, they like having a second set of ears there.” (Clinician 3).
**Patients**	
Socioeconomic disparities	“I don't have the technology and stuff to have any of that type of stuff. So it's only over the phone. Let me say this, I'm talking to you over the phone. I would much rather be coming in.” (Patient 7)
Racial/ethnic disparities	“You can feel better talking to your doctor… face to face than if you're just talking over the telephone. That's the way I was brought up thinking when you went to the doctor and you saw that person face to face. Now it might be old stuff, but that's just the way I see it nowadays.” (Patient 11) “And that's the only thing I have against the telephone doctor. I think the man should see you. Sometimes you can look at a person's face and tell something is wrong.” (Patient 33)

Clinicians reported disparities in quality of telehealth encounters among elders with hearing impairment. Clinician 6 said: “People whose elderly fingers shake too much to use these devices, or who have hearing [or] vision problems…[telehealth] magnifies disparities.” Clinician 13 explained that telehealth worsened quality of care, “especially with patients who are elderly and are hard of hearing. Over the phone, having a serious discussion…about dialysis is very challenging, and in some cases, delay[ed] getting them to have conversations.” However, because of the masking requirements in clinic, some patients with hearing impairments preferred video telehealth. Patient 1 explained: “I do a lot of lip reading because I have hearing loss and I can't lip read if everybody's got a mask on and that's true of my doctors too.”

Patients with limited English proficiency also struggled with telehealth, and clinician 12 explained: “Having conversations in telemedicine with an interpreter is a disaster. I can’t stand it…[It’s] very difficult to get the questions asked and answered, to double-check understanding. Patients who don’t speak English are at a disadvantage.” Clinicians mentioned the importance of health literacy for maintaining patient engagement and quality care in virtual visits. Clinician 9 remarked: “We have a subset of patients who just don't have any medical literacy. They have no idea what medicines they're taking. It's almost impossible to manage them [via telehealth].”

Patients of color were less satisfied with telehealth. They were more concerned about losing access to in-person visits, skeptical that clinicians could understand their chief complaints. Patient 26, who identified as Black, explained: “I need to see the doctor…her facial expressions. She should be able to see me and tell whether I'm okay with whatever she's saying or not. [You cannot] really do that over the telephone.” Patient 33, who also identified as Black, stated: “I certainly hope they don't make [telehealth] a habit….They can't really diagnose.” Patient 19, who identified as Native American, said: “[With telehealth] I feel like I'm on my own…I'm looking for help. And I wasn't getting it that much.”

## Discussion

This multisite qualitative study of telehealth experiences among older adults with advanced CKD, care partners, and clinicians identified key opportunities to improve patient-centered telehealth care for older adults with complex, chronic conditions by addressing perceived lower care quality due to inadequate physical examination, the loss of social connection and strained clinician-patient relationships, and disparities among older and structurally underserved populations. Clinicians were generally dissatisfied with telehealth, while most patients expressed more balanced perspectives, appreciating its convenience. Clinicians and patients emphasized loss of connection, and for clinicians, this led to dissatisfaction with telehealth. Patients shared this perspective to a lesser degree. Telehealth contributed to mistrust among some patients, and this was most consistently expressed among patients of color, who emphasized their preference to see the clinician in person and watch their body language. Our findings also underscore the benefits of telehealth for older adults, including convenience, perceived safety, care partner engagement, and improved understanding by clinicians of patients’ home environments. Future studies should examine strategies to promote patient-centered telehealth, given that patients’ and clinicians’ perceptions of telehealth are critical to widespread telehealth adoption.^21^

Although telehealth visits increased 154% from January through March 2020, compared with 2019,^32^ use plateaued in mid-April, and has subsequently declined.^33^ Our findings, including challenging social aspects and perceived lower-quality care, may help explain these declines. Quality of care posed a serious concern primarily for clinicians. CKD care, like the care of other chronic illnesses for older adults, largely relies on physical examination and laboratory tests that may not easily translate to virtual platforms. Concerns regarding inadequate home monitoring of vitals and failure to notice critical status changes undermined clinician and patient confidence in virtual visits. Similarly, concerns about the efficacy of telehealth physical examinations were the central challenges for teleoncology during the COVID-19 pandemic.^23^ To overcome this, alternating telehealth and in-person visits for older patients with chronic illness, as in the home dialysis telehealth policy in the 2018 Bipartisan Budget Act, may best balance participant burden and care quality concerns.^34^ Moreover, training in performing virtual physical examination is needed, future research should examine the efficacy and best practices for virtual physical examination given the rapid increase in telehealth for the management of chronic illnesses.

Care quality suffered among older patients who were less adept with technology, had limited access to video calls, and had minimal support.^35,36^ Many older patients were frustrated, reliant on care partners, and unable to engage meaningfully in visits. Similarly, patients with low socioeconomic status, limited health literacy, hearing impairment, or who were non-English speaking faced greater challenges engaging via telehealth. Patients of color were less satisfied with telehealth, many expressing mistrust in virtual interactions, unable to gauge whether the clinician was understanding their concerns. Our results extend prior survey findings demonstrating lower telehealth satisfaction among Black patients and more skepticism about confidentiality and the physical absence of a clinician.^16,23,37,38^ In CKD, people of color face greater barriers to accessing home-centric care, such as home dialysis and virtual health care visits.^39,40,41^ Structural inequalities further exacerbate the digital divide in access to stable internet connection, smartphones, and digital and health literacy. Our findings reinforce this but suggest that further examining the impact of perceived and experienced discrimination is important, as patients of color in our sample consistently expressed dissatisfaction and mistrust associated with telehealth encounters.^42^ To overcome these gaps, investment is needed, including distribution of technology and internet (eg, smartphones and tablets) within underserved communities, as suggested by some participants, and additional telehealth training for populations who need it most, including the elderly.

Our findings also offer insights into remedies to improve telehealth and suggest that telehealth may best supplement rather than supplant in-person visits for patients who are older and chronically ill. Clinicians suggested sharing detailed EMR summaries with patients and care partners and having advance practice providers follow-up after difficult conversations. Clinicians also suggested leaving patients time during the visit to process information and explicitly asking about emotions where nonverbal cues were unavailable. Implementing these strategies requires training clinicians and support staff in optimizing telehealth encounters while offering incentives and oversight tailored to improve patient satisfaction. Our findings clarify that care partner participation can improve care quality, especially for patients with limited health literacy, and was more easily achieved with telehealth than in-person.^3,43,44^

### Limitations

Limitations include recall bias and underrepresentation of Hispanic patients and non-English speakers, populations highly vulnerable and disproportionately affected by COVID-19 and CKD. Modest participation from 1 site may limit full appreciation of differences in telehealth delivery by site. Study strengths include a large geographically and racially diverse older sample that represents a spectrum of stakeholders.

## Conclusions

The findings of this study suggest a spectrum of telehealth satisfaction among older patients with CKD and care partners and found nephrologists tended to be less satisfied with telehealth. Training clinicians in virtual physical examinations, interspersing in-person visits, and interventions to mitigate disparities is needed.

## Notes

Supplement. **eAppendix 1.** Dual Anti-CTLA-4 and Anti-PD-1 blockade in Rare Tumors (DART) Trial Stakeholder Advisory Board **eAppendix 2.** Interview Script
