# Supplementary material for: Perceptions of Telehealth vs In-Person Visits Among Older Adults With Advanced Kidney Disease, Care Partners, and Clinicians
Source: JAMA Netw Open. 2021 Dec 6;4(12):e2137193. doi: 10.1001/jamanetworkopen.2021.37193 (PMC8649833; doi:10.1001/jamanetworkopen.2021.37193)
Supplement: Supplement. — eAppendix 1. Dual Anti-CTLA-4 and Anti-PD-1 blockade in Rare Tumors (DART) Trial Stakeholder Advisory Board eAppendix 2. Interview Script [file jamanetwopen-e2137193-s001.pdf]

Supplemental Online Content

Ladin K, Porteny T, Perugini JM, et al. Perceptions of telehealth vs in-person visits among older adults with advanced kidney disease, care partners, and clinicians. *JAMA Netw Open*. 2021;4(12):e2137193. doi:10.1001/jamanetworkopen.2021.37193

eAppendix 1. Dual Anti-CTLA-4 and Anti-PD-1 blockade in Rare Tumors (DART) Trial Stakeholder Advisory Board

eAppendix 2. Interview Script

This supplemental material has been provided by the authors to give readers additional information about their work.

© 2021 Ladin K et al. *JAMA Network Open.*

eAppendix 1. Dual Anti-CTLA-4 and Anti-PD-1 blockade in Rare Tumors (DART) Trial Stakeholder Advisory Board

DART Trial Stakeholder Advisory Board

1. **Geri Lynn Baumblatt, BA, MA**
   Co-Founder Difference Collaborative
2. **Megan Cheung, MSW**
   Associate Director, Clinical Director
   Greater Boston Chinese Golden Age Center
3. **Andrew K. Chin, MD**
   Professor, Health Science
   Division of Nephrology
   Department of Internal Medicine
   University of California Davis-Health
4. **Kelli Collins, MSW**
   Senior Patient Services Director
   National Kidney Foundation
5. **Renee Dupee**
   Deputy Director, Division of Kidney Health
   Centers for Medicare and Medicaid Services
6. **Catherine (Alicia) Georges, EdD, RN, FAAN**
   Professor and Chair,
   Department of Health Sciences, Human Services & Nursing
   Lehman College
   City University of New York
   National Volunteer President, AARP
7. **Rebecca N. Hutchinson, MD, MPH**
   Attending Physician
   Hospice and Palliative Medicine
   Maine Medical Center
   Faculty Scientist, Maine Medical Center Research Institute
8. **Richard Moscow**
   Patient Voice
9. **Trix Oakley**
   Care Partner Voice
10. **Scott Prushik, MD**
    Division of Vascular Surgery
    Saint Elizabeth's Medical Center
    Assistant Professor, Tufts University Medical School
11. **Shennon Wofford, MSW**
    CKD Care Coordinator Manager
    Dialysis Clinic, Inc.

© 2021 Ladin K et al. *JAMA Network Open.*

eAppendix 2. Interview Script

Clinicians will be asked about their experiences discussing treatment options with patients and care partners, with specific attention to their confidence discussing prognosis and CM, level of patient engagement in conversation, and perceptions of barriers, and facilitators of shared decision-making.

1. Tell me more about how COVID-19 has affected your practice.
    a. Timing of pandemic
    b. Type of visits (telehealth, in-person, hybrid, if a mix- who gets what?)
    c. Changes to staffing and education options (PA, options classes, dieticians, social work, etc.)
    d. Changes to patients on in-center HD
2. How has COVID-19 changed the way you are approaching discussion of treatment options as a result of COVID-19?
    a. Changes to education (if not answered above)
    b. Changes in treatment recommendations (changes in the default)
        i. Changes in treatment recommendations for specific patient groups- what characteristics/which groups are most likely to experience these changes?
    c. Changes in discussion of prognosis
    d. Changes in discussion of Conservative Management
3. Telehealth is providing a new platform to patients during these challenging times.
    a. How regularly to you use telehealth?
        i. Tell me more about your platform/logistics
    b. What is working well with telehealth?
    c. What is challenging about telehealth?
    d. How well do you think patients are able to engage in the encounter?
    e. How well are patients able to comprehend what you're telling them?
    f. How do you deal with emotional conversations over telehealth?
4. Who do you feel is being left behind in CKD treatment as a result of the shift to telehealth?
    a. Health literacy, language, mistrust, internet access, etc.
    b. Who do you think is being left behind as a result of COVID-19
    c. What do you think can be done to improve access for those who are being left behind? Could you provide some examples?
    d. Who is benefiting?
5. How do you think telehealth, and/or the COVID-19 pandemic have affected shared decision-making?

© 2021 Ladin K et al. *JAMA Network Open.*
